# A case report of severe pulmonary arterial hypertension after nivolumab, an IgG4 anti-PD1 monoclonal antibody

**DOI:** 10.1093/ehjcr/ytae222

**Published:** 2024-04-24

**Authors:** Yuanli Lei, Weijia Wang

**Affiliations:** Department of Hospital Medicine, Marshfield Clinic Health System, 1000 N Oak Ave, Marshfield, WI, USA; Department of Cardiology, Marshfield Clinic Health System, Marshfield, WI, USA

**Keywords:** Nivolumab, Immunotherapy, Pulmonary arterial hypertension, Right heart catheterization, Case report

## Abstract

**Background:**

Pulmonary hypertension has been increasingly reported in association with immunotherapy, but generally lacking invasive haemodynamic confirmation in literature. We present the first case of pulmonary arterial hypertension following nivolumab confirmed with invasive haemodynamic measurements.

**Case summary:**

A 65-year-old male with gastro-oesophageal adenocarcinoma developed progressive dyspnoea with exertion, decreasing exercise tolerance after receiving nivolumab for seven months. He was admitted with acute hypoxaemic respiratory failure after syncope at home. The patient was diagnosed with pulmonary arterial hypertension (PAH) with pre-capillary aetiology with right heart catheterization (RHC): mean pulmonary artery pressure 49 mmHg, pulmonary capillary wedge pressure 7 mmHg, and cardiac index 1.3 L/min/m^2^. Based on serial echocardiograms, the development of PAH appeared to be associated with nivolumab. The patient died of cardiac arrest 3 days after admission.

**Discussion:**

Progressive unexplained dyspnoea after receiving programmed cell death protein 1 monoclonal antibody should prompt clinicians to consider PAH and RHC.

**Learning points**

- Pulmonary hypertension has increasingly been reported to be associated with immunotherapy.
- Right heart catheterization should be promptly performed for diagnostic purposes when pulmonary hypertension is suspected in the context of immunotherapy to delineate differential diagnoses and devise treatment plans.

## Introduction

Nivolumab, a human IgG4 anti-programmed death (PD-1) monoclonal antibody, has been successfully utilized to treat different types of cancer. Immune-related adverse effects associated with nivolumab have been identified, including pneumonitis, hepatitis, neurotoxic effects, and thyroiditis.^1^ Recently, studies have suggested an association between PD-1/PD-L1 monoclonal antibodies and pulmonary arterial hypertension (PAH).^2,3^ However, the published literature has mostly used radiological data as a surrogate for diagnosis of pulmonary hypertension instead of invasive haemodynamics. We present a case of PAH diagnosed by right heart catheterization (RHC) associated with nivolumab.

## Summary figure

**Table:** 

Time	Event
Eight months prior	Diagnosed with gastro-oesophageal junction cancer Baseline echocardiogram showed normal biventricular function Chemotherapy and nivolumab were started
Six months prior	Echocardiogram after 2nd dose of nivolumab showing right heart dysfunction
Four weeks prior	Noticing dyspnoea with minimal exertion
One week prior	The last dose of nivolumab was administered (12 doses in total)
Admission	Admission after syncope
Day 3 of admission	Right heart catheterization: mean pulmonary artery pressure of 45 mmHg, pulmonary venous capillary wedge pressure of 7 mmHg, and pulmonary vascular resistance of 15.7 Wood units (Fick method)
Four hours after heart catheterization	Pulseless electrical activity, cardiac arrest

## Case presentation

A 65-year-old Caucasian male with a past medical history of stage IV moderately differentiated gastro-oesophageal junction adenocarcinoma diagnosed eight months ago presented with a witnessed syncope for 2 min at home. He received systemic chemotherapy with FOLFOX regimen (oxaliplatin, leucovorin, and 5-fluorouracil) since diagnosis. Nivolumab was started 7.5 months ago. He received nivolumab 240 mg every 2 weeks for a total of 12 doses with no significant side effects reported. Restaging workup 4 months before presentation showed decreased tumour size.

The patient had no history of pulmonary diseases. He reported progressive dyspnoea with walking to the bathroom for four weeks before presentation. Vital signs were heart rate 85 beats per minute, blood pressure 128/70 mmHg, and oxygen saturation 90% on 5 L/min supplemental oxygen.

**Figure 1:**
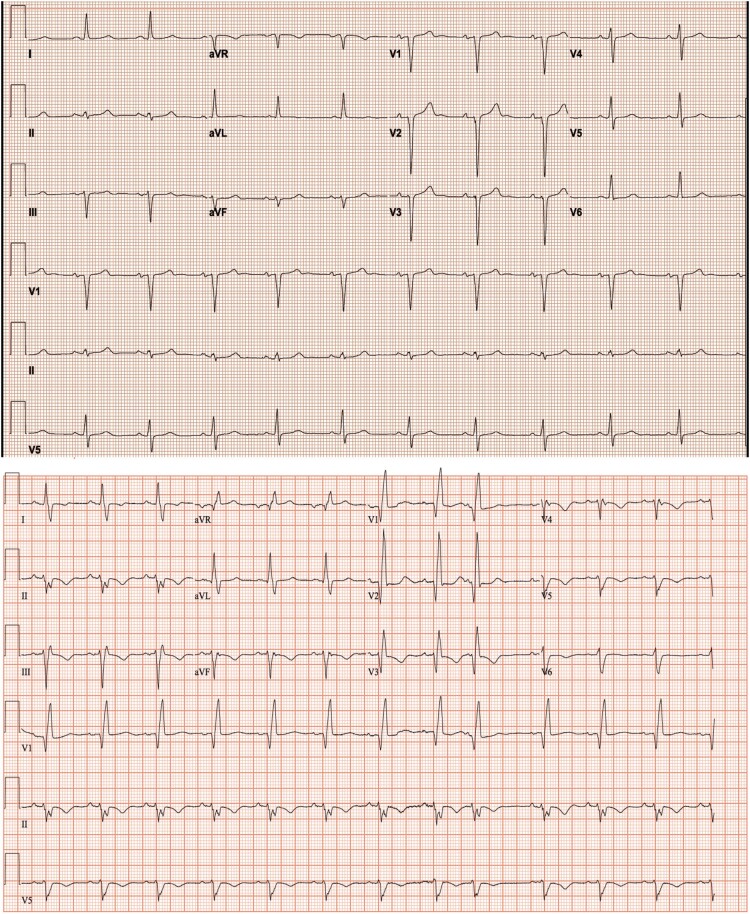
Baseline ECG obtained before nivolumab use (upper). ECG obtained on presentation after syncope (lower), showing reverse QRS transition on precordial leads, and right bundle branch block.

**Figure 2:**
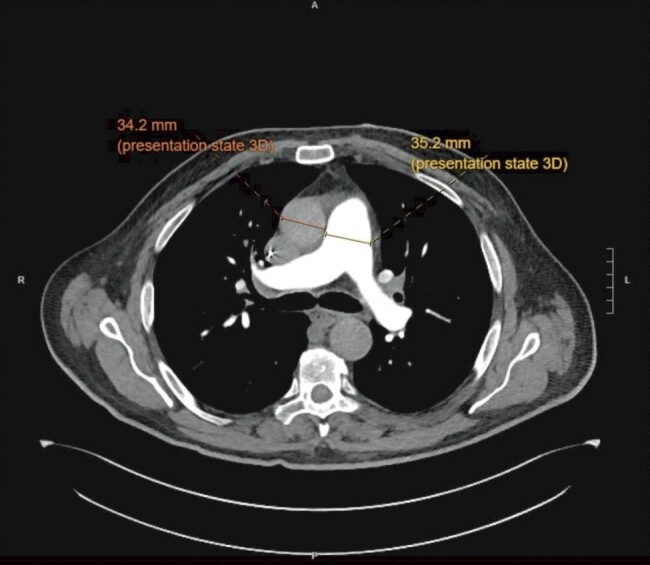
CT image showing the patient’s enlarged pulmonary artery (diameter 35.2 mm) compared with the aorta (diameter 34.2 mm).

Physical examination revealed clear lungs but bilateral leg pitting oedema. Electrocardiogram showed right ventricular strain pattern (*Figure 1*). High-sensitive troponin I levels were negative. B-type natriuretic peptide was 115 pmol/L (normal range < 9.5 pmol/L). CT pulmonary angiogram showed no pulmonary embolism or lung disease (*Figure 2*). His oxygen requirement improved to 2 L/min after diuresis. A transthoracic echocardiogram showed left ventricular ejection fraction (LVEF) of 29%, severely enlarged right ventricle (RV) (base diameter 6.5 cm, mid-cavity 4.6 cm) with reduced systolic function (fractional area change < 25%), D shaped septum in systole and diastole, moderate tricuspid regurgitation, and severe elevated pulmonary artery systolic pressure of 74 mmHg.

**Figure 3:**
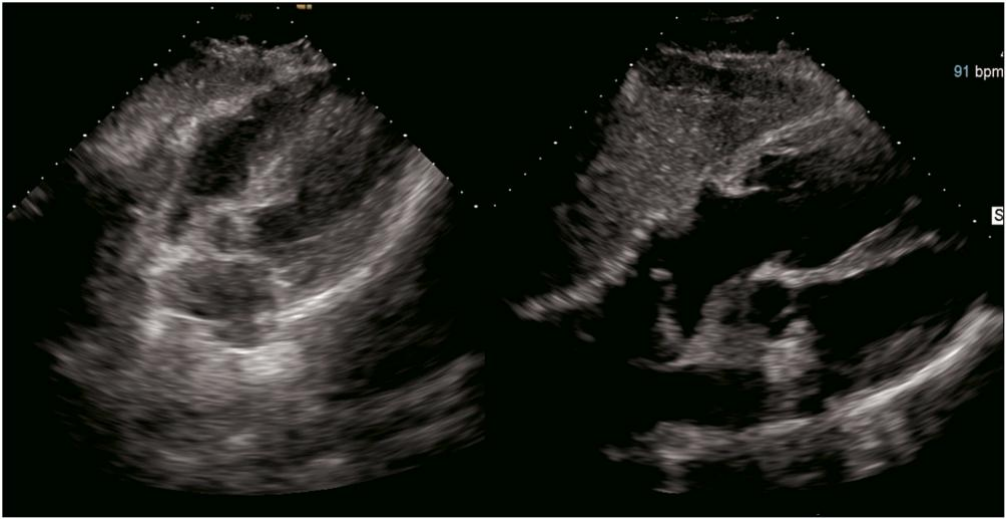
Echocardiograms (subcostal view) obtained before immunotherapy (left) and after admission (right) to demonstrate RV dilation.

An echocardiogram eight months ago (before systemic chemotherapy or nivolumab was started) showed an LVEF of 50%, normal RV size (base diameter 3.8 cm) and systolic function, and no tricuspid regurgitation. An echocardiogram six months ago (after cycle 2 of systemic chemotherapy and the second dose of nivolumab) showed an LVEF of 50%, borderline dilated RV (base diameter 4.0 cm) but normal systolic function, and trivial tricuspid regurgitation (*Figure 3*).

The coronary angiogram showed no obstruction. Left heart catheterization showed LV end-diastolic pressure of 6 mmHg (normal range 3–8 mmHg). Right heart catheterization showed mean pulmonary artery pressure of 45 mmHg (normal range < 25 mmHg), pulmonary venous capillary wedge pressure (PCWP) of 7 mmHg (normal range ≤ 12 mmHg), and pulmonary vascular resistance (PVR) of 15.7 Wood units (Fick method) (normal range ≤ 3 Wood units) (*Table 1*).

**Table 1:** Haemodynamic measurements during right and left heart catheterization

Parameter	Measured values	Normal range
PCWP (mmHg)	7	≤12
PA (mmHg; systolic/diastolic/mean)	72/36/49	15–25/6–12/<25
PA oxygen saturation (%)	30	
RV (mmHg; systolic/diastolic)	81/15	10–25/0–10
RA (mmHg; m)	15/11/9	0–5
Aorta (mmHg; systolic/diastolic/mean)	115/87/99	
Aorta oxygen saturation (%)	83% on room air	
LV (mmHg; systolic/diastolic)	123/6	
Cardiac output (L/min)	2.7	>5
Cardiac index (L/min/m^2^)	1.3	>2.4
PVR (Wood units; dyn·s/cm^5^)	15.7; 1258	≤3; ≤240

The patient had pulseless electrical activity arrest while in the restroom 4 h after cardiac catheterization. The patient was pronounced dead after resuscitation failed.

This patient experienced progressive exertional dyspnoea over one month with quick deterioration to syncope, acute hypoxaemic respiratory failure, and ultimately pulseless electrical activity arrest. This most likely represents an aggressive and fast-progressing form of PAH.

## Discussion

This is the first report of PAH following nivolumab confirmed with invasive haemodynamic measurements. Durvalumab has also previously been reported to be associated with PAH diagnosed via RHC.^4^ Currently, these are the only two published cases reporting an association between immunotherapy and PAH using invasive haemodynamic measurements. The ratio of pulmonary artery diameter over aorta diameter using CT angiogram and RV longitudinal strain on echocardiogram were utilized as indirect measurements of mean pulmonary artery pressure in other cases.^2,3^ Elevated pulmonary artery pressure is not equivalent to a diagnosis of PAH, as the latter can only be used to describe a pre-capillary aetiology causing elevated mean pulmonary artery pressure (>20 mmHg), and elevated PVR (>2 Wood units).^5^ Right heart catheterization is the gold standard to diagnose PAH by measuring both pulmonary artery pressure and wedge pressure. It is imperative to differentiate PAH from other groups of pulmonary hypertension as treatment options vary drastically.

PD-1 on T cells inhibits the activating signals from T-cell receptors. PD-1/PD-L1 interaction may lead to the inhibition of lymphocyte activation and proliferation, facilitating the expansion of regulatory T cells. Therefore, such an interaction limits tissue destruction during prolonged and uncontrolled inflammation.^6^ In animal models, regulatory T cells were noted to directly increase PGI_2_ and IL-10 production in cardiopulmonary circulation, and thus protect rats from developing pulmonary hypertension. Blocking the PD-1/PD-L1 pathway removes such protection.^7^ In humans, regulatory T cells and their subset in peripheral blood were noticed to be activated and are significantly higher in PAH patients when compared with healthy control.^6,8^ These data suggested that inhibition of PD-1/PD-L1 signalling in patients may increase the risk of PAH development, mediated through the regulatory T-cell pathway.

The aetiology of reduced LVEF and cardiac index (meeting cardiogenic shock criteria) of this patient was unclear. Cardiotoxicity from nivolumab could not be ruled out. In chronic LV dysfunction, pulmonary vasculature remodelling can occur with long-standing increased pulmonary venous pressure, resulting in a ‘persistent’ high PVR and pulmonary hypertension despite LV offloading.^9,10^ Left heart failure might have contributed to a combined form of PAH in this patient as provocative testing with normal saline infusion was not performed. In a medication safety report database, immune checkpoint inhibitor myocarditis cases were reported at a median of 27 days (range, 5–155 days) after immunotherapy initiation, with 76% of the cases occurring in the first 6 weeks of treatment.^11^ This patient developed heart failure later (7 months after receiving nivolumab). Stress-induced cardiomyopathy is another differential. Regardless of the aetiology for reduced LVEF, the significantly elevated PVR and pulmonary artery pressure with a normal PCWP suggests that PAH was the major contributor to his symptoms.

This patient had no history of pulmonary parenchymal disease. His hypoxaemic respiratory failure is acute-onset, rather than chronic, as seen in group 3 pulmonary hypertension.

The patient’s systemic chemotherapy FOLFOX regimen is not associated with pulmonary veno-occlusive disease.^12,13^ In the reported case of PAH associated with durvalumab, the patient was diagnosed with PAH associated with systemic lupus erythematosus/Sjogren’s syndrome overlap induced by PD-L1 inhibitor therapy due to the presence of autoantibodies, and the constellation of her symptoms. The patient also suffered several severe autoimmune side effects from immunotherapy. It was unclear whether the connective tissue disease was *de novo* or durvalumab unmasked her subclinical disease.^4^ Our patient, on the contrary, did not report autoimmune adverse effects from nivolumab throughout his cancer treatment. It is impossible to distinguish whether nivolumab or cancer itself triggered PAH. As gastro-oesophageal malignancy is not known to be associated with pulmonary hypertension, we speculate that nivolumab was more likely to be the culprit. His serial echocardiograms confirmed the temporal association between PAH and nivolumab, suggesting a causative effect. Should such causative effects establish with more evidence in the future, we believe that stopping nivolumab should be considered in addition to other standard treatments for PAH.

In summary, we report a case of PAH associated with nivolumab with invasive haemodynamic confirmation. Progressive unexplained dyspnoea after receiving PD-1 monoclonal antibody should prompt clinicians to consider PAH and RHC.

## Data Availability

The data underlying this article are available in the article.

## Lead author biography

Yuanli Lei is currently practicing hospital medicine. He graduated from Zhejiang University Medical School in China, and then completed Internal Medicine Residency at Houston Methodist Hospital in Houston, TX.

**Consent:** The authors confirm that written consent for submission and publication of this case report has been obtained from the patient in line with COPE guidance.

**Funding:** None declared.
